# Mental Health before and during the COVID-19 Pandemic: The Role of Partnership and Parenthood Status in Growing Disparities between Types of Families

**DOI:** 10.1177/00221465221109195

**Published:** 2022-07-15

**Authors:** Nicole Hiekel, Mine Kühn

**Affiliations:** 1 Max Planck Institute for Demographic Research, Rostock, Germany

**Keywords:** COVID-19 pandemic, family types, gender inequality, mental health inequalities

## Abstract

This study investigates mental health inequalities by family type and gender during the COVID-19 pandemic in Germany. Using data from the German Family Panel, we compared three dimensions of mental health (i.e., self-reported stress, exhaustion, and loneliness) one year before the pandemic and in spring 2020. First, two-parent families emerged as a vulnerable group, as the levels of stress and exhaustion they reported during the pandemic converged with those experienced by single parents. Second, a gender gap emerged during this global health crisis, with women, and particularly mothers, reporting significantly worse mental health compared to men in the same family type. Our findings underline the substantive value of studying mental health inequality from a multidimensional perspective and over time. Based on these findings, we urge policy makers to consider more seriously the disproportionate burdens that families, and women in particular, have been carrying due to the pandemic both directly and indirectly.

There is increasing evidence that the COVID-19 pandemic is exacerbating a wide range of preexisting social and economic inequalities. This may also be the case for mental health disparities. A rapidly growing body of COVID-19-related research has demonstrated that the pandemic had adverse effects on people’s mental health through rapid increases in stress and anxiety. These effects were triggered by the health threat of the virus, the lack of social integration and support, the difficulties people faced in accessing support services, and the economic downturn (Adams-Prassl et al. 2020; Bierman and Schieman 2020; Gabor et al. 2020; Gassman-Pines, Ananat, and Fitz-Henley 2020; Hart and Han 2020; Westrupp et al. 2020). Given the consistent evidence that before the pandemic, mental health had been unequally distributed across the population based on partnership (Umberson and Thomeer 2020) and parenthood status (Nomaguchi and Milkie 2020), the efforts to “flatten the curve” may have further contributed to growing disparities in the mental health levels of different types of families. If so, this exacerbation of mental health inequalities across types of families may be considered one of the unintended negative consequences of public health interventions in societies lacking herd immunity.

Like in many other countries affected by the COVID-19 pandemic, a key component of the governmental response in early 2020 in Germany was the implementation of far-reaching social-distancing measures aimed at slowing down the spread of the virus. These measures presented structural challenges that made it difficult for individuals to satisfy their human need for social belonging and integration. Research on mental health during the pandemic is needed to better understand the potential negative effects of the lack of social integration and support during this period on people’s feelings of social isolation and distress (Bierman and Schieman 2020).

In this article, we use a multidimensional concept of mental health and study how the dimensions of stress, exhaustion, and loneliness varied by family status—differentiated by partnership status and parenting status—before and during the COVID-19 pandemic and how these associations varied between men and women.

The public health interventions that were implemented early in the pandemic suddenly cut off parents from important sources of support. Most notably, educational and other facilities, like child care centers and schools, were closed, which had implications for parents’ efforts to reconcile paid employment and care work. The empirical evidence presented in this study on the growing inequalities in mental health in the wake of the pandemic based on parenthood status and gender can be used by policy makers to identify potential policy channels for designing and implementing effective recovery strategies.

We analyze data from the Panel Analysis of Intimate Relationships and Family Dynamics (Pairfam). Pairfam is a large, nationally representative, prospective study of German adults (Huinink et al. 2011). In spring 2020, a COVID-19 supplement was fielded that collected information on the effects of the COVID-19 pandemic on individuals’ private lives and personal relationships. These data have large methodological advantages over data from studies using convenience samples because they enable us to compare mental health disparities by family status and gender before and during the pandemic using four subpopulations representing the most prevalent family types in Germany.

This article contributes to the literature on the effects of the COVID-19 pandemic on mental health inequalities in several ways. Our study is the first on this topic that compares individuals in different family types. Whereas the existing studies have mainly focused on single subgroups, such as adolescents and parents (Gabor et al. 2020; Janssen et al. 2020; Zoch, Bächmann, and Vicari 2021), we consider singles, childless couples, partnered parents, and single parents. This approach enables us to assess the mental health of men and women with a similar partnership status, parenthood status, or both and to examine mental health disparities by family type between two points in time (i.e., one year before the pandemic and during the pandemic). We thus provide empirical evidence of the emergence of new inequalities in mental health between men and women in particular types of families that may be rooted in their experiences during the pandemic. Studying in a quasi-natural experiment how the external shocks of the pandemic and the policy responses to it have shaped the social context in which individuals respond not only adds to our understanding of how the current coronavirus pandemic has contributed to the growing disparities in mental health between different social groups; it also provides us with a better understanding of the general mechanisms that underlie the complex relationships between partnership status, parenthood, and mental health.

## Background

### Prepandemic Inequalities in Mental Health by Family Status

#### Partnership status and mental health

Family scholars have long studied the relationship between marital status and mental health. Empirical research that has predominantly focused on comparisons between married and nonmarried people has consistently shown that married people have better health outcomes than their unmarried counterparts (Carr and Springer 2010). The beneficial effects of marriage have mainly been attributed to two mechanisms: selection and causation. The *selection perspective* emphasizes that healthier people are more likely to marry and remain married (positive health selection). The *causation perspective* offers two explanations for these effects: (a) The *resource perspective* argues that marriage provides economic and psychological resources that enhance the psychological well-being of the partners and that reduce their psychological distress, whereas (b) the *crisis perspective* argues that married people are healthier than people who have experienced a union dissolution because divorce is associated with stress, which is harmful to mental health (Amato 2000). The recent availability of longitudinal data on married and unmarried people has enabled scholars to investigate the underlying mechanisms of the marriage effect, and most have found evidence for both the selection and the causation perspectives (Goldman 2001).

Unmarried cohabitation is an increasingly prevalent and socially accepted practice and has become an inherent feature of contemporary relationship careers (Sassler and Lichter 2020). Therefore, we argue in favor of theoretical developments that seek to incorporate unmarried cohabitation into models predicting mental health levels by partnership status. Cohabitation is among the key elements of the ongoing evolution of the institution of marriage, not least because more couples than ever are cohabiting before they get married. In contexts in which cohabitation is more prevalent among the socioeconomically disadvantaged, cohabiting unions often dissolve quickly and transitions to marriage from cohabitation are less frequent. This pattern has, for example, been observed in the United States (Lichter and Qian 2008). At the same time, cohabitation has been found to offer fewer mental health benefits than marriage (Brown 2000; Marcussen 2005). Over the past decade, scholars have accumulated scientific evidence that the benefits of marriage and cohabitation are converging as cohabitation becomes more common and more socially accepted (Sassler and Lichter 2020). In countries where cohabitation is less socially selective and the social norms allow individuals to make choices regarding cohabitation that traditionally applied only to marriage, no differences in the mental health of married and cohabiting individuals have been found (Stavrova and Fetchenhauer 2015). Hence, in these contexts, cohabiters benefit from marriage-like resources. The vast majority of German cohabiters marry their cohabiting partner (Hiekel 2014). Thus, the high degree of “instability” in cohabiting unions in Germany can be explained by the finding that cohabiting unions are 5 times as likely to transition to marriage than they are to dissolve (Hiekel and Fulda 2018). Therefore, in the context of the present study, we assume that theoretical arguments about the selection and resource perspectives can be applied to both married and cohabiting individuals (Hiekel 2014).

However, union dissolution and widowhood are associated with adverse changes in mental health, as a very large body of literature has shown (for reviews, see Carr and Springer 2010; Holm, Berland, and Severinsson 2019; Onrust and Cuijpers 2006). Studies that have investigated the relationship between experiencing a divorce, a separation, or the death of a partner and having poor mental health have shown that this association can be partly explained by selection (i.e., that individuals with poor health are more prone to experience these life events; Wade and Pevalin 2004). To explain these adverse effects of divorce or widowhood on mental health, the *stress model* is often used. Amato (2000) distinguished between partnership loss as a stressful event from which individuals tend to recover and partnership loss as a process that initiates the accumulation of life strains that negatively affect individuals’ mental health in the long run, such as economic hardship, social isolation, and single parenthood.

#### Parental status and mental health

There is a large and diverse body of research investigating parents’ psychological and emotional well-being. Some studies have suggested that parents report higher levels of well-being (e.g., greater life satisfaction or happiness) than adults without children, whereas other studies have shown that parents have lower levels of well-being (e.g., lower life satisfaction, more depression, anxiety, and stress) than nonparents (for reviews, see Nelson, Kushlev, and Lyubomirsky 2014; Umberson, Pudrovska, and Reczek 2010).

Recent work has explained these mixed findings using the framework of the *demands-rewards perspective*, which argues that parenthood brings both demands (i.e., physical, mental, and financial efforts) and rewards (i.e., achievement of parenting goals) to the lives of adults (Nomaguchi and Milkie 2020), and thus concludes that parenting elicits a “mixed bag” of emotions (Musick, Meier, and Flood 2016). Negraia and Augustine (2020) unpacked this mixed bag and found that compared to nonparents, the parents of young children experience both more negative emotions (i.e., stress and fatigue) and more positive emotions (i.e., more happiness and less sadness).

However, whether the costs and rewards of parenthood are balanced seems to depend on the parents’ social status and stage of parenting (Nelson et al. 2014; Ruppanner, Perales, and Baxter 2019; Simon and Caputo 2019) as well as the social policy context (Glass, Simon, and Andersson 2016). There is consistent evidence that the parents of younger children experience higher levels of psychological distress than the parents of older children and the childless (Evenson and Simon 2005). Such findings are often explained by the extreme time constraints, the reduced relationship quality between the parents, the child care demands, and the increased financial responsibilities that the parents of young children often experience (Negraia and Augustine 2020). These effects tend to be greater among socioeconomically disadvantaged social groups, who have fewer resources to cope with these demands (Nomaguchi and Milkie 2020).

Furthermore, a cross-national comparison uncovered a gap in mental health between parents depending on their social policy context, with the disadvantages associated with parenthood being smallest in countries that provide high levels of family support. Thus, the results of this comparison suggest that the levels of support a nation provides to parents to help them raise their children can have positive effects on the parents’ mental health (Glass et al. 2016).

Single parents tend to experience greater parenting stress, which can negatively affect their mental health, and these effects may, in turn, intersect with the effects of gender and socioeconomic status. Given that they often have to provide the entire household income, single parents, and particularly single mothers, face higher levels of stress due to economic pressures, intensive caregiving responsibilities, and work–family conflicts. There is evidence that compared to partnered mothers, single mothers report less happiness and more sadness, stress, and fatigue associated with parenting (Kühn 2018; Meier et al. 2016).

Based on these theoretical and empirical considerations, we expect to find that when we compare the initial (i.e., the prepandemic) mental health of various groups, partnered individuals had better mental health than unpartnered individuals, couples without children had better mental health than couples with children, and partnered parents had better mental health than single parents (Hypothesis 1).

### Inequalities in Mental Health by Family Status During the Pandemic

During the COVID-19 pandemic, individuals with a partner in the household could compensate for the lack of social contacts outside the home to some extent. However, the sudden social isolation associated with the pandemic may have negatively affected the mental well-being of unpartnered individuals in particular. For some of these individuals, the lockdown may have interrupted a coping strategy of cultivating a wide social support network of friends and acquaintances to compensate for the absence of a partner. Moreover, the lockdown may have prevented these individuals from maintaining social contacts outside of their own household that helped them in pursuing their goal of finding a partner. For single individuals without children in the household, the lack of social life may have led to their leisure time becoming a source of boredom, loneliness, and worry.

Due to the legally binding restrictions on contact in public spaces and orders to “stay home” during the lockdown, people’s access to (physical) social interactions and support from members of different households was greatly reduced. The regular access people had to face-to-face social interactions with other adults not living in the same household, which was once taken for granted, vanished from one day to the next. Colleagues were working from home or were required to adhere to strict physical distancing practices while at the workplace. Associations and clubs that previously brought people together to play sports, listen to music, maintain traditions, or cultivate other hobbies canceled their activities. Friends and family members were strongly advised to maintain physical distance from each other due to a lack of knowledge about how the virus was transmitted.

For many parents with dependent children, the closure of day care centers and schools made it much more difficult for them to juggle their many responsibilities, including participating in the labor market and caring for their children. Employed single parents and parents in dual-earner households were most affected by these closures. Many of these parents were forced to either to cut their working hours or, when possible (especially if they were in a high-skilled job), to combine working from home with looking after their children (Andrew et al. 2020; Huebener et al. 2021). Previous studies have shown that child care instability is stressful for parents, and especially for single mothers, who tend to have difficulties coping with care disruptions (Pilarz and Hill 2017). Thus, it is likely that the pandemic led to new levels of *role strain*, a term used to describe “the felt difficulty in fulfilling role obligations” (Goode 1960:483). Role strain tends to occur when parents experience pressure to engage in contradictory activities and thus experience conflicts of time, place, or resource allocation. Family relationships are the most immediate and persistent context in which role strain may negatively affect individual well-being because formal withdrawal from social obligations is difficult, and informal withdrawal from these obligations may lead to both feelings of distress and the imposition of feelings of guilt and sanctions by family members.

Based on these theoretical considerations, we expect to observe that differences in mental health by family status became more pronounced during the lockdown between unpartnered and partnered individuals, between couples with children and couples without children, and between single-parent and two-parent families (Hypothesis 2).

### Gender Differences in the Link between Family Status and Mental Health

Gender differences in the association between family status and mental health are often attributed to mothers having more role obligations within the family, which decreases the beneficial effects of partnership and parenthood on women’s mental health. The types of roles that women tend to adopt within the family include performing unpaid care work, emotional work, and housework for the benefit of their partner and their children. As these tasks are considered time-consuming and onerous, mothers may have a greater mental load and be more prone to frustration than fathers. Previous studies have clearly shown that the costs of parenthood are more severe for mothers than for fathers (Nomaguchi, Milkie, and Bianchi 2005; Ruppanner et al. 2019), with fathers spending more time than mothers engaged in activities that bring joy and are associated with less stress, such as play and leisure activities (Musick et al. 2016). Furthermore, most single parents are women, and single mothers tend to have fewer resources and less support to buffer their parenting (and other sources of) stress than men (Burgard and Ailshire 2013; Negraia, Augustine, and Prickett 2018). While the beneficial effects of marriage and the negative effects of union dissolution on mental health seem to affect women and men similarly, the symptoms they experience tend to differ. While unmarried women report having more depressive symptoms than married women, unmarried men often increase their alcohol use (Simon 2002).

We expect to find that the initial (i.e., prepandemic) mental health of mothers was lower than that of fathers (Hypothesis 3). Furthermore, we use an explorative approach to investigate gender differences in partnership status and mental health.

The increase in the mental health disadvantages of women during the lockdown may be linked to the clear gender inequalities in paid work, housework, and care work, all of which were strongly affected by the measures the government imposed to tackle the COVID-19 pandemic. The existing evidence on the consequences of the COVID-19 lockdown suggests that while men increased their overall contributions to housework and care work from low initial levels, the lockdown was not the great equalizer but, rather, reaffirmed or even magnified the existing gender inequalities within couples with children (Czymara, Langenkamp, and Cano 2021; Hank and Steinbach 2020; Kreyenfeld and Zinn 2021; Möhring et al. 2020; Rodríguez Sánchez, Fasang, and Harkness 2021). Therefore, it is likely that after the onset of the pandemic, women’s unpaid workload further increased from already high levels because during the spring lockdown, families were spending more time at home than they were before the pandemic. Thus, during the lockdown, women were spending even more hours on child care, home schooling, meal preparation, cleaning, and grocery shopping. On a different note, there is initial evidence for Germany suggesting that the pandemic has had gender-specific labor market consequences because more women than men have been engaged in stressful and risky work on the frontlines because of their overrepresentation in system-relevant occupations (Adams-Prassl et al. 2020; Bünning, Hipp, and Munnes 2020; Zoch et al. 2021). We thus expect to find clear gender differences in mental health, with mothers having lower levels of mental health than fathers (Hypothesis 4). The differences in the mental health levels of unpartnered women and unpartnered men are investigated in an explorative fashion.

## Data and Methods

The Panel Analysis of Intimate Relationships and Family Dynamics (Pairfam) is a large, nationally representative, prospective study of German adults that has been running since 2008–2009 (Brüderl et al. 2021; Huinink et al. 2011). Currently, there are 11 annual regular panel waves that provide information on three German birth cohorts (1971–1973, 1981–1983, 1991–1993, and since Wave 11, 2001–2003). For our analyses of prepandemic mental health inequalities, we used data from Wave 11, which were collected roughly one year before the pandemic (November 2018 to March 2019).

The fieldwork for the regular 12th panel wave had to be paused during the pandemic in March 2020. Between May 19 and July 13, 2020, an additional optional 15-minute web-based survey covering the effects of the COVID-19 pandemic on the respondents’ private lives and personal relationships was fielded among panel members who were part of the gross sample for Wave 12. A total of 3,154 (of the eligible 9,640) respondents completed the so-called COVID-19 survey. The majority of the data had been collected by mid-June.

The youngest Pairfam cohort was born between 2001 and 2003 and were thus between 17 and 20 years old when the COVID-19 survey data were collected. Respondents below age 18 received a somewhat different set of questions tailored to their specific life situation. Moreover, 97% of this group were living with their parents; only 18 were living with a partner, and just 2 had a child. Thus, our theoretical arguments linking parental status and partnership status to mental health disparities could not be applied to this population. By limiting our analysis to respondents of the three older Pairfam birth cohorts (1971–1973, 1981–1983, and 1991–1993), our analytical sample was reduced from 3,154 to 2,163 respondents. For the same theoretical reason mentioned previously, we further excluded respondents who were living with their parents but without a partner or children (n = 140) or with nonkin only (n = 110). Finally, inclusion in the sample was conditioned on having valid responses to items that addressed the three dimensions of mental health before and during the pandemic and other variables included in the models. This further reduced our sample by 48. Our final analytical sample comprised 1,865 women and men.

We performed a set of robustness analyses that addressed potential bias in our estimates arising from nonparticipation in the COVID-19 survey. The results of these analyses may be found in Tables S5 through S7 in the online version of the article. We applied inverse probability weights to our regression models as a robustness analysis. To obtain these weights, we predicted participation in the optional COVID-19 survey in 2020 using logit regression models, including a set of characteristics known to predict panel attrition. Based on this model, we estimated inverse probability weights for each respondent and applied these to the models presented in the main article. The conclusions drawn from these weighted robustness analyses are virtually identical to the results based on unweighted models presented here.

### Measurements

Pairfam collects information on respondents’ *mental health* along different dimensions and implements items from established scales that tend to be modified by shortening the original number of items through tapping into the construct or by unifying the scale length. We decided to focus on those dimensions of mental health for which we could obtain measurements both before and during the pandemic. This resulted in three dimensions of mental health: levels of stress, lack of energy, and loneliness. All of these dimensions were measured by asking respondents, “How have you been feeling, for the most part, during the past four weeks?” The response categories ranged from 1 (does not at all apply) to 5 (applies absolutely).

To measure stress, we used three items designed to capture the feelings of being “stressed,” “overburdened,” and “under pressure.” These items are part of a stress scale developed by Fliege et al. (2001). To measure lack of energy, we used two items that assessed whether the respondents were feeling “active” and “full of energy.” These items are part of the psychological state scale developed by Abele-Brehm and Brehm (1986). We reversed the items so that larger values indicated lower mental health, consistent with the other two dimensions of mental health studied here. To assess levels of loneliness, we used one item that measured the extent to which respondents were “feeling alone.” The item originally stemmed from the UCLA loneliness scale developed by Russell, Peplau, and Cutrona (1980). A second item was introduced in the COVID-19 survey that captured the extent to which the respondents were “feeling lonely.” This item was adapted from the Psychological Adjustment to the COVID-19 Pandemic Study (Schmidt et al. 2021).

We obtained three mean scores for the mental health dimensions *stress*, *lack of energy*, and *loneliness* by summing answers to up to three questions for each dimension and dividing that number by the number of items for each dimension. A higher mean score indicates worse mental health in that dimension. For the multivariate results, we transformed the mean scores to have a mean of 0 and a standard deviation of 1.

Based on the information respondents provided about with whom they were coresiding, we identified their partnership and parenthood status and distinguished the four most common *family types* in Germany: namely, living single, living with a partner and without child(ren), living with a partner and child(ren), and living without a partner and with child(ren).

We validated the distribution of family types in our analytical sample and compared it to official statistics data. To do so, we drew on data from the 2020 German Mikrozensus (MZ), an annually conducted official statistical survey using a representative sample of 1% of the German population and households (Federal Office of Statistics 2020). Because respondents were required by law to participate, we considered the MZ an appropriate source to validate our family type distribution. The distribution of family types in our analytical sample corresponded to that of the population in Germany. In both the MZ and our analytical sample (AS), two-parent families were the most prevalent family type (42% in MZ vs. 51% in AS), followed by childless couples (30% in MZ vs. 25% in AS). Individuals living alone (20% in MZ vs. 17% in AS) and single parents (8% in MZ vs. 8% in AS) were the least prevalent family types.

Education and cohort membership are potential confounding variables in the association between family type and mental health because they select people into different family types (Amato 2010; Sharma 2015) and mental health outcomes (Evans et al. 2019; Schneider 2012). In addition, compared to the regular pairfam panel, the COVID-19 survey had more female than male panel members, slightly more university-educated participants, and more members of the younger panel cohorts.^1^ We added these variables as control variables in our models.

### Analytical Approach

We ran a set of ordinary least squares regression models with the standardized mental health score for each of the three indicators as the dependent variable. To test Hypotheses 1 and 2 regarding whether the association between family type and mental health had changed from the period before to the period during the pandemic, we included the respondents’ family type and gender, which we identified as the two key explanatory variables needed to answer our research question. In the model predicting that there were mental health disparities between family types during the pandemic, we additionally included each respondent’s corresponding prepandemic mental health score to account for individual floor and ceiling effects. To test Hypotheses 3 and 4 regarding gender differences in the association between family type and mental health before and during the pandemic, we additionally included an interaction term between each family type and gender in each of the models addressing one of the three mental health indicators.

## Results

### Descriptive Findings

Table 1 presents the distribution of each of the nontransformed mental health dimensions by family type one year prior to the pandemic and during the pandemic. Overall, reported levels of stress were higher prior to the pandemic than during the pandemic. By contrast, for the dimensions of lack of energy and loneliness, overall levels of mental health were lower during the pandemic than before the pandemic. Single parents reported lower levels of mental health in all dimensions than respondents with other family types both before and during the pandemic. The distribution of our control variables, educational attainment, gender, and cohort, reflected the overrepresentation of highly educated respondents (57%) and women (60%) in our analytical sample that we discussed previously, but not that of members of the panel’s youngest birth cohort (24%).

**Table 1.** Descriptive Results for 1,865 Men and Women in Four Different Family Types.

	Single Parent	Two-Parent Family	Single	Childless Couple	Total Sample
**Family type, % (*n*)**	7.56 (141)	50.83 (948)	16.62 (310)	24.99 (466)	100.00 (1,865)
**Gender, % (*n*)**					
Women	9.98 (111)	51.98 (578)	13.85 (154)	24.19 (269)	59.62 (1,112)
Men	3.98 (30)	49.14 (370)	20.72 (156)	26.16 (197)	40.38 (753)
**Mental health** ^a^					
Stress					
Prepandemic	3.43	3.03	3.05	3.06	3.07
During pandemic	3.07	2.92	2.64	2.63	2.81
Lack of energy					
Prepandemic	2.79	2.74	2.82	2.82	2.78
During pandemic	3.19	2.94	2.97	2.96	2.96
Loneliness					
Prepandemic	2.52	1.67	2.62	1.64	1.88
During pandemic	2.46	1.79	2.66	1.96	2.01
**Educational degree, %**					
All levels of secondary	53.19	42.93	41.29	42.92	43.43
Tertiary	46.81	57.07	58.71	57.08	56.57
**Birth cohort, %**					
1991–1993	7.09	4.64	49.03	52.79	24.24
1981–1983	40.43	56.56	35.81	34.12	46.33
1971–1973	52.48	38.71	15.16	13.09	29.44

*Source:* Panel Analysis of Intimate Relationships and Family Dynamics Wave 11 and COVID-19-Survey.

a Mean scores based on multiple indicators tapping into the dimensions *stress*, *lack of energy*, and *loneliness*; in the regression models, these have been included as *z*-standardized mean scores.

### Multivariate Findings

#### Prepandemic mental health discrepancies by family type

**Figure 1.**
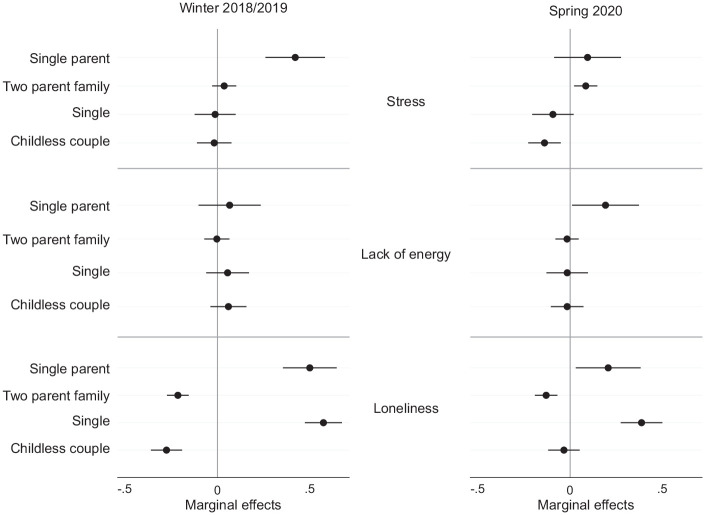
Mental Health by Family Type before and during the Pandemic (n = 1,865). *Note:* Models control for gender, university degree (yes/no), and birth cohort. Models on 2020 data additionally control for prepandemic level of mental health in corresponding dimension. *Source:* Panel Analysis of Intimate Relationships and Family Dynamics Wave 11 (2018–2019) and COVID-19 Survey.

The left panel of Figure 1 plots the results of three regression models predicting mental health disparities based on three indicators of mental health between respondents in different types of families before the pandemic, expressed as marginal effects. On the *x*-axis, the predicted levels of stress, lack of energy, and loneliness are plotted. The value 0 refers to the sample mean (which is set to 0 and a standard deviation of 1 due to a *z*-standardization of the sum score), with negative values indicating lower levels of mental health compared to the sample mean and positive values indicating higher levels of mental health compared to the sample mean. On the *y*-axis, the four different family types are distinguished. We present the results of the full regression models (the β coefficients) in Table S1 in the online version of the article.

One year prior to the pandemic, single parents clearly had levels of stress that were higher than average. Their predicted stress levels were close to half a standard deviation above the sample mean, while the predicted stress levels of individuals in any other family type clustered around the sample mean, and overlapping confidence intervals suggest that there were no significant differences between them. Single respondents exhibited the predicted levels of lack of energy (i.e., levels that were higher than the sample average). However, because all family types clustered around the sample’s mean value for lack of energy and the confidence intervals were overlapping, we conclude that there was no systematic variation in exhaustion by family type in our sample one year prior to the pandemic. Nonetheless, levels of loneliness clearly differed by family type. The findings suggest that the extent to which respondents felt lonely one year prior to the pandemic differed by partnership status rather than by parenthood status. The predicted levels of loneliness among single parents and single individuals were more than half a standard deviation above the sample mean, with no significant differences between the two groups. Individuals in a two-parent family and individuals in a childless union reported similar levels of loneliness that were slightly below the sample mean.

From these analyses, we conclude that first, systematic mental health disparities by family type prior to the pandemic do not reflect the mental health gradient that Hypothesis 1 suggested. To the extent that we observed worse mental health among individuals in certain family types than in others, the mental health disadvantage appeared to be linked to partnership status rather than to parenthood status, as indicated by the higher levels of stress and loneliness found among single parents and by the higher levels of loneliness observed among singles without children in the household.

#### Pandemic mental health discrepancies by family type

During the pandemic, the predicted levels of stress among single-parent families and two-parent families converged. As a result, a gap in stress levels emerged between family types characterized by parenthood status that was not evident prior to the pandemic. Regarding the predicted levels of lack of energy, all family types were clustered around the sample mean, with one exception: Single parents had a significantly increased risk of feeling exhausted compared to all other family types that was not apparent one year earlier. Variation in the levels of loneliness across family types exhibited a nearly identical pattern both during and before the pandemic. Overall, however, we observed a starker pattern of convergence toward the sample mean for the predicted levels of loneliness among all family types. Single parents and single respondents exhibited predicted levels of loneliness that were significantly above the sample average and that were significantly higher than those of any family type in which a partner was present.

Taken together, the findings on mental health disparities by family type during the pandemic appear to represent a continuation of the mental health inequalities at the intersection of parenthood and partnership status that were already apparent prior to the pandemic. We found that single-parent families represented a family type that was persistently more vulnerable than other family types to having worse mental health outcomes, as indicated by their above-average levels of stress, lack of energy, and loneliness. Nevertheless, our analyses also uncovered a few aspects of mental health inequalities that suggested that certain vulnerabilities increased during the pandemic and may have even reached unprecedented levels. For example, the finding that a gap in stress levels opened up between parents and nonparents supports the assumption made in Hypothesis 2 that group disparities increased between the periods before and during the pandemic. Thus, partnered parents emerged as a social group in which individuals had become increasingly vulnerable to having above-average levels of stress. Regarding loneliness, our findings indicated that there was an overall decrease in variation by family type, while the overall pattern remained largely unchanged. Although a population-level increase in feelings of loneliness during the pandemic was reported in the descriptive results, the multivariate analyses presented here provide evidence that this increase was largely equally distributed across the family types studied.

#### Gender differences in mental health by family type before and during the pandemic

**Figure 2.**
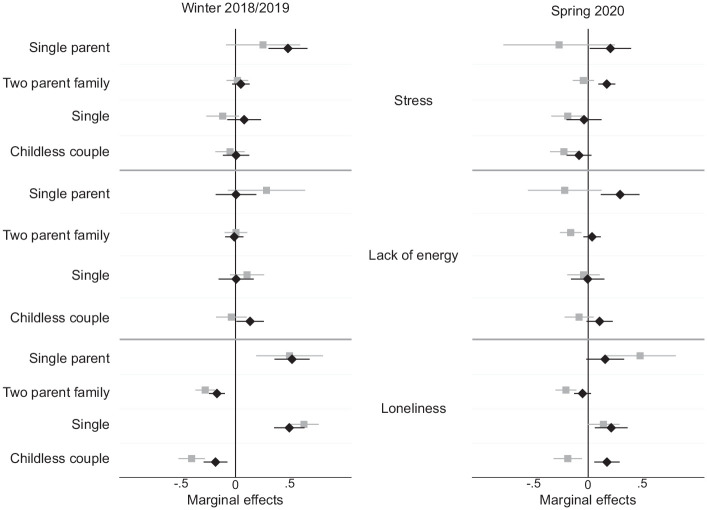
Differences between Women (diamonds) and Men (squares) in Mental Health by Family Type before and during the Pandemic (n = 1,865). *Note:* Models control for gender, university degree (yes/no), and birth cohort. Models on 2020 data additionally control for prepandemic level of mental health in corresponding dimension. *Source:* Panel Analysis of Intimitate Relationships and Family Dynamics Wave 11 (2018-2019) and COVID-19 Survey.

In this section, we present the results of our analyses regarding the gender gap in mental health by family type before and during the pandemic. Figure 2 plots the results of the ordinary least squares regression models predicting the gender differences in mental health between respondents in different types of families before the pandemic (left panel) and in spring 2020 (right panel), expressed as marginal effects (the full regression tables can be found in Table S2 in the online version of the article).

While before the pandemic, none of the associations between family types and mental health outcomes differed significantly between men and women, our results revealed that during the pandemic, a gender gap in mental health emerged among parents and particularly between partnered mothers and partnered fathers. Mothers in two-parent families reported significantly higher levels of stress, lack of energy, and loneliness than their male counterparts. Partnered mothers’ predicted levels of stress and exhaustion were significantly higher than the sample mean, while their male counterparts’ predicted levels of stress and exhaustion were around or even below the average. The results comparing single mothers and single fathers pointed to an increase in the gender gap that preceded the onset of the pandemic. However, given the small number of single fathers in our sample (as indicated by the large confidence interval around the estimate), the statistical power of this analysis was too low to allow for any firm conclusions to be drawn.

Taken together, the findings on the prepandemic measures of mental health suggest that the gender differences predicted in Hypothesis 3 could not be validated by our data. One year prior to the pandemic, men and women exhibited similar levels of stress, lack of energy, and loneliness depending on their parenthood status rather than on their partnership status. The findings on the gender differences in mental health levels during the pandemic supported Hypothesis 4. In spring 2020, mothers—and especially mothers living with a partner and children—exhibited higher predicted levels of stress, lack of energy, and loneliness than fathers in the same types of families did.

Because our finding that there was an emerging gap in mental health between mothers and fathers in two-parent families was striking, we ran a further set of explorative analyses to better understand where this gap may have come from. We report the outcomes of these analyses in detail in Tables S3 and S4 and the corresponding Figures S1 and S2 in the online version of the article. The results indicated that unequally distributed child care responsibilities and gendered work–family conflicts may explain some of the mental health gap between mothers and fathers during the pandemic. For stress and loneliness, but not for a lack of energy, we found a clear mental health disadvantage among mothers when their youngest child was preschool age but smaller (primary school age) or nonexistent (secondary school age) differences between the mothers and fathers of school-aged children (see Figure S1 in the online version of the article). For stress but not for energy and loneliness, we found clear evidence that the gender gap among two-parent families could be explained by the mothers of preschool-aged children who were working from home reporting significantly higher stress levels than their female counterparts who were not working from home (see Figure S2 in the online version of the article). These findings suggest that in spring 2020, the mental health of mothers was more negatively affected by work–family conflicts than that of fathers.

## Discussion

This study set out to investigate whether the increase in social inequalities during the COVID-19 pandemic that has been observed for a wide range of social and economic factors also applies to dimensions of mental health. For German men and women of working ages who belonged to the four most common family types, we studied three dimensions of mental health: levels of stress, lack of energy, and loneliness. We compared mental health disparities by family type and gender one year before the pandemic and during the first infection wave in spring 2020, when public health measures were in place that imposed unprecedented limits on social support and integration for most of the German population.

Our analyses showed that there were no systematic disparities across all dimensions of mental health between types of families. Thus, this study underlined the empirical and substantive benefits of studying mental health outcomes from a multidimensional perspective. To the extent that we found that the mental health of individuals in certain family types was worse during than before the pandemic, the mental health disadvantage appeared to be linked to partnership status rather than to parenthood status.

Our analyses of mental health inequalities by family type during the pandemic generated three main findings. First, we identified mental health disparities by family type that we did not observe prior to the pandemic. On the stress dimension, parents, regardless of their partnership status, were found to have significantly worse mental health than nonparents. Thus, parenthood status was identified as a key component in mental health inequalities during the pandemic. Earlier studies have shown that constant demands for resilience can produce overwhelming stress (Mikolajczak et al. 2018), which might explain why parents who were juggling paid employment, care work, and education for their children during a global health crisis reported having worse mental health than individuals without children.

Second, our study revealed that the mental health disadvantage of single parents persisted and grew larger for the exhaustion dimension. Even before the pandemic, single parents reported having above-average levels of stress and loneliness. The observation that single parents face an elevated risk of having poor mental health outcomes is well established in the literature and has largely been attributed to their high and continuous exposure to the challenges associated with being the main caregiver and wage provider (Avison, Ali, and Walters 2007; Crosier, Butterworth, and Rodgers 2007).

We were admittedly surprised that our analyses did not reveal an ever-increasing gap in stress levels between single parents and the other family groups during the pandemic. A study based on Australian data showed that although single mothers were the most severely time-stressed group in the prepandemic period, they experienced the most relief in terms of time stress early in the pandemic (Craig and Churchill 2021). The authors attempted to explain this result by speculating that single mothers, who were more likely than partnered mothers to be working full-time, may have been disproportionately relieved of work expectations when kindergartens and schools were closed. We argue in a similar vein and suggest that single parents may have been better able than partnered parents to activate an already established informal support network when institutional child care support was not available. In Germany in spring 2020, so-called emergency child care (i.e., access to nurseries, kindergartens, and afternoon schools), which was initially only available to so-called essential workers, was increasingly made available to single parents and thus may have provided some relief to these families.

Nevertheless, the finding that single parents had higher levels of exhaustion than all other family types is likely a direct consequence of the pandemic amplifying the multiple strains with which sole caregivers and wage providers with family obligations were already dealing. Given that single mothers and their children are at disproportionally high risk of poverty and mental health and economic resilience are interrelated in complex ways, there is an urgent need to provide societal support in navigating the pandemic to these vulnerable families.

Third, while we showed that the stratification in loneliness by family type slightly converged toward the sample mean, this finding obscures the increase in overall levels of loneliness observed across our sample (i.e., everyone was feeling lonelier than they were before). Given the evidence that persistent feelings of loneliness increase the risk of developing serious mental health problems (Mushtaq et al. 2014; Nuyen et al. 2020), these findings point to a general population health concern that should be given greater attention by policy makers seeking to mitigate the effects of nonpharmaceutical public health interventions aimed at slowing down the spread of infection by restricting contact.

This study also addressed gender differences in the mental health disparities between family types. One year prior to the pandemic, men and women exhibited similar levels of stress, lack of energy, and loneliness, and the gender differences within family types were not significant. However, in spring 2020, women and men, and particularly mothers and fathers, reported significantly different levels of mental health. The results revealed that mothers had significantly higher levels of stress, lack of energy, and loneliness than fathers did and that this gap was particularly striking for partnered mothers. We would have expected to find that partnered mothers showed greater resilience than single mothers. For instance, a study that focused on levels of parenting-related exhaustion during the 2020 lockdown in Italy found that mothers experienced greater levels of exhaustion than fathers, particularly when they were single, had children with special needs, had a larger number of children, or had younger children (Marchetti et al. 2020).

Thus, partnered mothers in Germany have emerged as a newly vulnerable group during the current global health crisis. Our supplementary analyses showed that in two-parent families, mothers’ (but not fathers’) predicted levels of stress were higher the younger the coresident children were. Moreover, mothers (but not fathers) who worked remotely during the spring lockdown reported the highest levels of stress when their coresiding children were preschool age. We interpret these explorative analyses as evidence that mothers suffered more than fathers from having to balance child care, employment, and, most likely, additional domestic work during the pandemic in Germany. The widening gender gap in mental health found among parents is in line with the findings of U.S.-based research (Adams-Prassl et al. 2020) and of studies that focused on German families (Huebener et al. 2021; Zoch et al. 2021). The pandemic has revealed that many families are juggling a male-oriented employment life course and unreliable child care on their own. This is a symptom of the individualization of structural social problems that comes with a heavy cost (i.e., the deterioration in the mental health of mothers, which could have negative consequences for the well-being of their children). The stressors associated with the pandemic that we argue are contributing to mental health disparities by family type and gender are still accumulating and are likely to increase further in the future.

To ensure that families of all types recover from the effects of the pandemic, policy makers must acknowledge and address the disproportionate burdens that mothers have carried in navigating this global crisis. Thus, policies aimed at helping families recover from setbacks they experienced during the pandemic need to embed a gender-sensitive approach. Beyond providing for targeted measures, such as increased public spending on child care and early education, policies must embed a gender lens into all aspects of governance to avoid reinforcing existing gender inequalities. The collection and analysis of data that reflect the differences in how the pandemic affected women and men, and specifically mothers and fathers, will provide a solid empirical basis for crafting gender-inclusive frameworks and plans that take into account gender inequalities in households, in the labor market, and in society at large. Later this year, Pairfam panel data for fall/winter 2021 will become available and will thus allow for further monitoring of how different types of families—and mothers compared to fathers—fared in the second year of the pandemic.

Our study is not without limitations. Due to education-based panel attrition, highly educated individuals were overrepresented in our sample. High socioeconomic status individuals were undeniably more likely than their lower socioeconomic status counterparts to have more resources before and during the pandemic. Thus, they may have been better able to adhere to the nonpharmaceutical public health measures implemented in spring 2020 and to mitigate the negative consequences associated with these interventions. It is therefore possible that the mental health disparities by family type in the general population are larger than those reported in this study. However, the key findings presented here—namely, that there is a growing gap in mental health between parents and nonparents that seems to be largely driven by the emergence of mental health disparities between mothers and fathers—may be applicable to women of higher socioeconomic status in particular. These women may have been at greater risk of experiencing sharp increases in work–family conflicts during the pandemic. Highly educated women tend to be more attached to the labor market. At the same time, the traditional distribution of gender roles in German culture makes women highly dependent on extra-household child care to reconcile paid and unpaid work. Employers’ efforts to support their staff by helping them reconcile child care and paid work were often limited to facilitating working from home and offering flexible schedules—both of which applied to the jobs of high socioeconomic status women and tended to have more adverse effects on women (mothers) than on men (fathers; Lott 2020). As our supplementary analyses on partnered mothers and fathers showed, the mental health of fathers during the pandemic did not vary by the age of their youngest child or by whether they were working from home, whereas mothers of younger children, and particularly those who were working from home during the spring lockdown, had the highest stress levels.

Our categorization of individuals into only four family types does not fully reflect the heterogeneity of families in Germany. First, we deliberately ignored nonresident partners. We felt that this approach was justified by the theoretical considerations we presented previously because particularly during the lockdowns, which imposed restrictions on physical contact, coresidence was a stronger than normal precondition for securing support and social integration. Second, we ignored nonresident children. However, given the age range of our sample, most of these children were coresiding with an ex-partner of the respondents. We believe that the main implications of the exclusion of these children are that we may have underestimated the care burdens of the respondents in our sample and particularly those of respondents assigned to family types characterized by the absence of coresident children. Third, we deliberately ignored household members other than each respondent’s partner and children. The small number of families in our sample that included other coresident members and the diversity of the types of relationships between the respondents and these additional household members made it impossible for us to arrive at a subdivision that was more nuanced and was still meaningful. As a robustness check, we reanalyzed all data while excluding these cases, and the results suggest that the findings presented here are not biased by their inclusion (we report the outcomes of these analyses in Tables S5 through S7 in the online version of the article). Another limitation of our study was that the respondents’ mental health was measured at only two points in time. Therefore, we were not able to identify individual changes in mental health by considering unobserved heterogeneity. However, the differences in mental health we found between family types highlight the existence of vulnerable groups, which should be investigated in further research.

A final challenge we faced was potential bias due to selection into participation in the optional COVID-19 survey. We ran a set of robustness analyses that addressed potential bias in our estimates arising from nonparticipation in the COVID-19 survey. We applied inverse probability weights to our regression models. The conclusions drawn from these weighted robustness analyses were virtually identical to the results based on the unweighted models presented here.

## Supplemental Material

sj-docx-1-hsb-10.1177_00221465221109195 – Supplemental material for Mental Health before and during the COVID-19 Pandemic: The Role of Partnership and Parenthood Status in Growing Disparities between Types of Families Click here for additional data file. Supplemental material, sj-docx-1-hsb-10.1177_00221465221109195 for Mental Health before and during the COVID-19 Pandemic: The Role of Partnership and Parenthood Status in Growing Disparities between Types of Families by Nicole Hiekel and Mine Kühn in Journal of Health and Social Behavior

## Notes

1. https://www.pairfam.de/fileadmin/user_upload/redakteur/publis/Dokumentation/TechnicalPapers/Technical_Paper_15.pdf.

## References

[bibr1-00221465221109195] Abele-Brehm Andrea Brehm Walter . 1986. “Zur Konzeptualisierung und Messung von Befindlichkeit: Die Entwicklung der “Befindlichkeitsskalen”(BFS) [On the Concept and Measurement of State of Mind: The Development of the State of Mind Scales].” Diagnostica 32(3):209–28.

[bibr2-00221465221109195] Adams-Prassl Abi Boneva Teodora Golin Marta Rauh Christopher . 2020. “The Impact of the Coronavirus Lockdown on Mental Health: Evidence from the U.S.” Working Paper 2020-030, Human Capital and Economic Opportunity Working Group. https://www.repository.cam.ac.uk/handle/1810/334492.

[bibr3-00221465221109195] Amato Paul R. 2000. “The Consequences of Divorce for Adults and Children.” Journal of Marriage and Family 62(4):1269–87.

[bibr4-00221465221109195] Amato Paul R. 2010. “Research on Divorce: Continuing Trends and New Developments.” Journal of Marriage and Family 72(3):650–66.

[bibr5-00221465221109195] Andrew Alison Cattan Sarah Dias Monica Costa Farquharson Christine Kraftman Lucy Krutikova Sonya Phimister Angus Sevilla Almudena . 2020. “How Are Mothers and Fathers Balancing Work and Family Under Lockdown?” Institute for Fiscal Studies. https://ifs.org.uk/publications/14860. 10.1111/1475-5890.12240 PMC7753283 33362314

[bibr6-00221465221109195] Avison William R. Ali Jennifer Walters David . 2007. “Family Structure, Stress, and Psychological Distress: A Demonstration of the Impact of Differential Exposure.” Journal of Health and Social Behavior 48(3):301–17. 10.1177/002214650704800307 17982870

[bibr7-00221465221109195] Bierman Alex Schieman Scott . 2020. “Social Estrangement and Psychological Distress before and during the COVID-19 Pandemic: Patterns of Change in Canadian Workers.” Journal of Health and Social Behavior 61(4):398–417. 33211540 10.1177/0022146520970190

[bibr8-00221465221109195] Brown Susan L. 2000. “The Effect of Union Type on Psychological Well-Being: Depression among Cohabitors Versus Marrieds.” Journal of Health and Social Behavior 41(3):241–55. 11011503

[bibr9-00221465221109195] Brüderl Josef Drobnič Sonja Hank Karsten Neyer Franz J. Walper Sabine Alt Philipp Borschel Elisabeth , et al. 2021. “The German Family Panel (pairfam).” GESIS Data Archive, Cologne ZA5678 Data File Version 12.0.0 [data set].

[bibr10-00221465221109195] Bünning Mareike Hipp Lena Munnes Stefan . 2020. “Erwerbsarbeit in Zeiten von Corona.” WZB Ergebnisbericht, Wissenschaftszentrum für Sozialforschung, Berlin. http://hdl.handle.net/10419/216101.

[bibr11-00221465221109195] Burgard Sarah A. Ailshire Jennifer A. 2013. “Gender and Time for Sleep among U.S. Adults.” American Sociological Review 78(1):51–69. 25237206 10.1177/0003122412472048 PMC4164903

[bibr12-00221465221109195] Carr Deborah Springer Kristen W. 2010. “Advances in Families and Health Research in the 21st Century.” Journal of Marriage and Family 72(3):743–61.

[bibr13-00221465221109195] Craig Lyn Churchill Brendan . 2021. “Unpaid Work and Care During Covid-19: Subjective Experiences of Same-Sex Couples and Single Mothers in Australia.” Gender & Society 35(2):233–43.

[bibr14-00221465221109195] Crosier Timothy Butterworth Peter Rodgers Bryan . 2007. “Mental Health Problems among Single and Partnered Mothers.” Social Psychiatry and Psychiatric Epidemiology 42(1):6–13. 17203237 10.1007/s00127-006-0125-4

[bibr15-00221465221109195] Czymara Christian S. Langenkamp Alexander Cano Tomás . 2021. “Cause for Concerns: Gender Inequality in Experiencing the COVID-19 Lockdown in Germany.” European Societies 23(Suppl. 1):68–81.

[bibr16-00221465221109195] Evans M. D. R. Kelley Jonathan Kelley S. M. C. Kelley C. G. E. 2019. “Rising Income Inequality during the Great Recession Had No Impact on Subjective Wellbeing in Europe, 2003–2012.” Journal of Happiness Studies 20(1):203–28.

[bibr17-00221465221109195] Evenson Ranae J. Simon Robin W. 2005. “Clarifying the Relationship between Parenthood and Depression.” Journal of Health and Social Behavior 46(4):341–58. 10.1177/002214650504600403 16433280

[bibr18-00221465221109195] Federal Office of Statistics. 2020. “Bevölkerung und Erwerbstätigkeit. Haushalte und Familien. Ergebnisse des Mikrozensus 2019.” [Population and Labor Force. Households and families. Results of the microcensus 2019] Fachserie 1 Reihe 4, Wiesbaden, Germany.

[bibr19-00221465221109195] Fliege Herbert Rose Matthias Arck Petra Levenstein Susan Klapp Burghard F. 2001. “Validierung des ‘Perceived Stress Questionnaire’ (PSQ) an einer Deutschen Stichprobe” [Validation of the “Perceived Stress Questionnaire”(PSQ) in a German Sample]. Diagnostica 47(3):142–52.

[bibr20-00221465221109195] Gabor Csikos Dr Törő Krisztina Mokos Judit Sándor Rózsa Éva Hadházi Andrea Kövesdi Rita Földi . 2020. “Examining Perceptions of Stress, Wellbeing and Fear among Hungarian Adolescents and Their Parents Under Lockdown during the COVID-19 Pandemic.” PsyArXiv. doi:10.31234/osf.io/feth3.

[bibr21-00221465221109195] Gassman-Pines Anna Ananat Elizabeth Oltmans Fitz-Henley John . 2020. “COVID-19 and Parent–Child Psychological Well-Being.” Pediatrics 146(4):1–9. 10.1542/peds.2020-007294 PMC7546085 32764151

[bibr22-00221465221109195] Glass Jennifer Simon Robin W. Andersson Matthew A. 2016. “Parenthood and Happiness: Effects of Work-Family Reconciliation Policies in 22 OECD Countries.” American Journal of Sociology 122(3):886–929. 10.1086/688892 PMC5222535 28082749

[bibr23-00221465221109195] Goldman Noreen . 2001. “Social Inequalities in Health Disentangling the Underlying Mechanisms.” Annals of the New York Academy of Sciences 954(1):118–39. 11797854

[bibr24-00221465221109195] Goode William J. 1960. “A Theory of Role Strain.” American Sociological Review 25(4):483–96.

[bibr25-00221465221109195] Hank Karsten Steinbach Anja . 2020. “The Virus Changed Everything, Didn’t It? Couples’ Division of Housework and Childcare before and during the Corona Crisis.” Journal of Family Research 33(1):99–114.

[bibr26-00221465221109195] Hart Jake Han Wen-Jui . 2020. “COVID-19 Experiences and Parental Mental Health.” Journal of the Society for Social Work and Research 12(2):283–302.

[bibr27-00221465221109195] Hiekel Nicole . 2014. The Different Meanings of Cohabitation across Europe. How Cohabiters View Their Unions and Differ in Their Plans and Behaviors. Amsterdam: Amsterdam University Press.

[bibr28-00221465221109195] Hiekel Nicole Fulda Barbara Elisabeth . 2018. “Love. Break up. Repeat: The Prevalence and Stability of Serial Cohabitation among West German Women and Men Born in the Early 1970s.” Demographic Research 39(30):855–70.

[bibr29-00221465221109195] Holm Anne Lise Berland Astrid Karin Severinsson Elisabeth . 2019. “Factors That Influence the Health of Older Widows and Widowers—A Systematic Review of Quantitative Research.” Nursing Open 6(2):591–611. 30918710 10.1002/nop2.243 PMC6419130

[bibr30-00221465221109195] Huebener Mathias Waights Sevrin Spiess C. Katharina Siegel Nico A Wagner Gert G . 2021. “Parental Well-Being in Times of Covid-19 in Germany.” Review of Economics of the Household 19:91–122. 33469413 10.1007/s11150-020-09529-4 PMC7808123

[bibr31-00221465221109195] Huinink Johannes Brüderl Josef Nauck Bernhard Walper Sabine Castiglioni Laura Feldhaus Michael . 2011. “Panel Analysis of Intimate Relationships and Family Dynamics (Pairfam): Conceptual Framework and Design.” Zeitschrift für Familienforschung 23(1):77–101.

[bibr32-00221465221109195] Janssen Loes H. C. Kullberg Marie-Louise J. Verkuil Bart van Zwieten Noa Wever Mirjam C. M. van Houtum Lisanne A. E. M. Wentholt Wilma G. M. Elzinga Bernet M. 2020. “Does the COVID-19 Pandemic Impact Parents’ and Adolescents’ Well-Being? An EMA-Study on Daily Affect and Parenting.” PloS One 15(10):e0240962. doi:10.1371/journal.pone.0240962. PMC7567366 33064778

[bibr33-00221465221109195] Kreyenfeld Michaela Zinn Sabine . 2021. “Coronavirus and Care: How the Coronavirus Crisis Affected Fathers’ Involvement in Germany.” Demographic Research 44(4):99–124.

[bibr34-00221465221109195] Kühn Mine . 2018. “Changes in Lone Mothers’ Health: A Longitudinal Analysis.” Pp. 323–38 in Lone Parenthood in the Life Course, edited by Bernardi L. Mortelmans D. Cham: Springer International Publishing.

[bibr35-00221465221109195] Lichter Daniel T. Qian Zhenchao . 2008. “Serial Cohabitation and the Marital Life Course.” Journal of Marriage and Family 70(4):861–78.

[bibr36-00221465221109195] Lott Yvonne . 2020. “Does Flexibility Help Employees Switch off from Work? Flexible Working-Time Arrangements and Cognitive Work-to-Home Spillover for Women and Men in Germany.” Social Indicators Research 151(2):471–94.

[bibr37-00221465221109195] Marchetti Daniela Fontanesi Lilybeth Mazza Cristina Di Giandomenico Serena Roma Paolo Verrocchio Maria Cristina . 2020. “Parenting-Related Exhaustion During the Italian COVID-19 Lockdown.” Journal of Pediatric Psychology 45(10):1114–23. 10.1093/jpepsy/jsaa093 PMC7665691 33068403

[bibr38-00221465221109195] Marcussen Kristen . 2005. “Explaining Differences In Mental Health between Married and Cohabiting Individuals.” Social Psychology Quarterly 68(3):239–57.

[bibr39-00221465221109195] Meier Ann Musick Kelly Flood Sarah Dunifon Rachel . 2016. “Mothering Experiences: How Single Parenthood and Employment Structure the Emotional Valence of Parenting.” Demography 53(3):649–74. 10.1007/s13524-016-0474-x PMC5497991 27150964

[bibr40-00221465221109195] Mikolajczak Moira Brianda Maria Elena Avalosse Hervé Roskam Isabelle . 2018. “Consequences of Parental Burnout: Its Specific Effect on Child Neglect and Violence.” Child Abuse & Neglect 80:134–45. 10.1016/j.chiabu.2018.03.025 29604504

[bibr41-00221465221109195] Möhring Katja Naumann Elias Reifenscheid Maximiliane Blom Annelies G. Wenz Alexander Rettig Tobias Lehrer Roni , et al. 2020. “Die Mannheimer Corona-Studie: Schwerpunktbericht zu Erwerbstätigkeit und Kinderbetreuung.” University of Mannheim. https://madoc.bib.uni-mannheim.de/55139/.

[bibr42-00221465221109195] Mushtaq Raheel Shoib Sheikh Shah Tabindah Mushtaq Sahil . 2014. “Relationship between Loneliness, Psychiatric Disorders and Physical Health? A Review on the Psychological Aspects of Loneliness.” Journal of Clinical and Diagnostic Research 8(9):WE01–4. 10.7860/JCDR/2014/10077.4828 PMC4225959 25386507

[bibr43-00221465221109195] Musick Kelly Meier Ann Flood Sarah . 2016. “How Parents Fare: Mothers’ and Fathers’ Subjective Well-Being in Time with Children.” American Sociological Review 81(5):1069–95.

[bibr44-00221465221109195] Negraia Daniela Veronica Augustine Jennifer March . 2020. “Unpacking the Parenting Well-Being Gap: The Role of Dynamic Features of Daily Life across Broader Social Contexts.” Social Psychology Quarterly 83(3):207–28.

[bibr45-00221465221109195] Negraia Daniela Veronica Augustine Jennifer March Prickett Kate Chambers . 2018. “Gender Disparities in Parenting Time across Activities, Child Ages, and Educational Groups.” Journal of Family Issues 39(11):3006–28.

[bibr46-00221465221109195] Nelson S. Katherine Kushlev Kostadin Lyubomirsky Sonja . 2014. “The Pains and Pleasures of Parenting: When, Why, and How Is Parenthood Associated with More or Less Well-Being?” Psycholgical Bulletin 140(3):846–95. 10.1037/a0035444 24491021

[bibr47-00221465221109195] Nomaguchi Kei Milkie Melissa A. 2020. “Parenthood and Well-Being: A Decade in Review.” Journal of Marriage and Family 82(1):198–223. 32606480 10.1111/jomf.12646 PMC7326370

[bibr48-00221465221109195] Nomaguchi Kei M. Milkie Melissa A. Bianchi Suzanne M. 2005. “Time Strains and Psychological Well-Being: Do Dual-Earner Mothers and Fathers Differ?” Journal of Family Issues 26(6):756–92.

[bibr49-00221465221109195] Nuyen Jasper Tuithof Marlous de Graaf Ron van Dorsselaer Saskia Kleinjan Marloes Ten Have Margreet . 2020. “The Bidirectional Relationship between Loneliness and Common Mental Disorders in Adults: Findings from a Longitudinal Population-Based Cohort Study.” Social Psychiatry and Psychiatric Epidemiology 55(10):1297–310. 10.1007/s00127-019-01778-8 31538206

[bibr50-00221465221109195] Onrust Simone A. Cuijpers Pim . 2006. “Mood and Anxiety Disorders in Widowhood: A Systematic Review.” Aging Mental Health 10(4):327–34. 10.1080/13607860600638529 16798624

[bibr51-00221465221109195] Pilarz A. R. Hill H. D. 2017. “Child-Care Instability and Behavior Problems: Does Parenting Stress Mediate the Relationship?” Journal of Marriage and Family 79(5):1353–68. 10.1111/jomf.12420 PMC5666338 29104315

[bibr52-00221465221109195] Rodríguez Sánchez Alejandra Fasang Anette Harkness Susan . 2021. “Gender Division of Housework During the COVID-19 Pandemic: Temporary Shocks or Durable Change?” Demographic Research 45(43):1297–316.

[bibr53-00221465221109195] Ruppanner Leah Perales Francisco Baxter Janeen . 2019. “Harried and Unhealthy? Parenthood, Time Pressure, and Mental Health.” Journal of Marriage and Family 81(2):308–26.

[bibr54-00221465221109195] Russell Dan Peplau Letitia A. Cutrona Carolyn E. 1980. “The Revised UCLA Loneliness Scale: Concurrent and Discriminant Validity Evidence.” Journal of Personality and Social Psychology 39(3):472–80. 10.1037//0022-3514.39.3.472 7431205

[bibr55-00221465221109195] Sassler Sharon Lichter Daniel T. 2020. “Cohabitation and Marriage: Complexity and Diversity in Union-Formation Patterns.” Journal of Marriage and Family 82(1):35–61.

[bibr56-00221465221109195] Schmidt Andrea Brose Annette Kramer Andrea C. Schmiedek Florian Witthöft Michael Neubauer Andreas B. 2021. “Dynamic Relations among COVID-19-Related Media Exposure and Worries During the COVID-19 Pandemic.” Psychology and Health. 10.1080/08870446.2021.1912345 33886394

[bibr57-00221465221109195] Schneider Simone M. 2012. “Income Inequality and Its Consequences for Life Satisfaction: What Role do Social Cognitions Play?” Social Indicators Research 106(3):419–38. doi:10.1080/08870446.2021.1912345.

[bibr58-00221465221109195] Sharma Andy . 2015. “Divorce/Separation in Later-Life: A Fixed Effects Analysis of Economic Well-Being by Gender.” Journal of Family and Economic Issues 36(2):299–306.

[bibr59-00221465221109195] Simon Robin W. 2002. “Revisiting the Relationships among Gender, Marital Status, and Mental Health.” American Journal of Sociology 107(4):1065–96. 10.1086/339225 12227382

[bibr60-00221465221109195] Simon Robin W. Caputo Jennifer . 2019. “The Costs and Benefits of Parenthood for Mental and Physical Health in the United States: The Importance of Parenting Stage.” Society and Mental Health 9(3):296–315.

[bibr61-00221465221109195] Stavrova Olga Fetchenhauer Detlef . 2015. “Married and Cohabiting Parents’ Well-Being: The Effects of a Cultural Normative Context across Countries.” Journal of Social and Personal Relationships 32(5):601–32.

[bibr62-00221465221109195] Umberson Debra Pudrovska Tetyana Reczek Corinne . 2010. “Parenthood, Childlessness, and Well-Being: A Life Course Perspective.” Journal of Marriage and Family 72(3):612–29. 10.1111/j.1741-3737.2010.00721.x PMC3159916 21869847

[bibr63-00221465221109195] Umberson Debra Thomeer Mieke Beth . 2020. “Family Matters: Research on Family Ties and Health, 2010 to 2020.” Journal of Marriage and Family 82(1):404–19. 10.1111/jomf.12640 PMC8048175 33867573

[bibr64-00221465221109195] Wade Terrance J. Pevalin David J. 2004. “Marital Transitions and Mental Health.” Journal of Health and Social Behavior 45(2):155–70. 10.1177/002214650404500203 15305757

[bibr65-00221465221109195] Westrupp Elizabeth Bennett Clair Berkowitz Tomer S. Youssef George Toumbourou John Tucker Richard Andrews Fiona Evans Subhadra Teague Samantha Karantzas Gery . 2020. “Child, Parent, and Family Mental Health and Functioning in Australia During COVID-19: Comparison to Pre-Pandemic Data.” PsyArXiv. doi:10.31234/osf.io/ydrm9. PMC8379590 34417875

[bibr66-00221465221109195] Zoch Gundula Bächmann Ann-Christin Vicari Basha . 2021. “Who Cares When Care Closes? Care-Arrangements and Parental Working Conditions During the COVID-19 Pandemic in Germany.” European Societies 23(1):576–88.

