# Supplementary material for: Mental Health before and during the COVID-19 Pandemic: The Role of Partnership and Parenthood Status in Growing Disparities between Types of Families
Source: J Health Soc Behav. 2022 Jul 15;63(4):594–609. doi: 10.1177/00221465221109195 (PMC9716053; doi:10.1177/00221465221109195)
Supplement: sj-docx-1-hsb-10.1177_00221465221109195 – Supplemental material for Mental Health before and during the COVID-19 Pandemic: The Role of Partnership and Parenthood Status in Growing Disparities between Types of Families [file sj-docx-1-hsb-10.1177_00221465221109195.docx]

**Journal** of **Health**

and **Social Behavior**

OFFICIAL JOURNAL OF THE AMERICAN SOCIOLOGICAL ASSOCIATION

**ONLINE SUPPLEMENT**

**to article in**

Journal of Health and Social Behavior

**Mental Health before and during the COVID-19 Pandemic: The Role of Partnership and Parenthood Status in Growing Disparities between Types of Families**

**Nicole Hiekel**

*Max Planck Institute for Demographic Research*

**Mine Kühn**

*Max Planck Institute for Demographic Research*

*Table S1*. Results from linear regression models for n=1,865 women and men predicting stress, lack of energy and loneliness before and during the Covid-19 pandemic by family type

|  | Stress | | Lack of Energy | | Loneliness | |
| --- | --- | --- | --- | --- | --- | --- |
| Variable | Before pandemic | Spring 2020 | Before pandemic | Spring 2020 | Before pandemic | Spring 2020 |
| Single parent | .46***  (.10) | .23* (.10) | .01  (.10) | .21*  (.10) | .84***  (.10) | .24* (.10) |
| Two parent family | .06  (.06) | .22***  (.06) | -.06  (.06) | -.00 (.06) | .07  (.06) | -.10 (.06) |
| Single | .01  (.07) | .05  (.07) | -.00  (.07) | .00  (.07) | .92*** (.07) | .42***  (.07) |
| Childless couple (Reference) |  |  |  |  |  |  |
| Women (vs. men) | .08  (.05) | .19*** (.05) | .00  (.05) | .18*** (.05) | .10*  (.04) | .18*** (.04) |
| Pre-pandemic level of mental health |  | .29***  (.02) |  | .29*** (.02) |  | .29***  (.02) |
| University educated (vs. not) | .13**  (.05) | .01  (.04) | -.09*  (.05) | .00  (.04) | -.02  (.04) | -.07  (.04) |
| Birth cohort  1991-93 | .10  (.07) | -.13*  (.06) | -.01  (.07) | .04 (.06) | .07  (.06) | .08  (.06) |
| 1981-83 (Reference) |  |  |  |  |  |  |
| 1971-73 | -.13*  (.05) | -.17***  (.05) | -.14*  (.06) | -.14**  (.05) | -.04  (.05) | -.07  (.05) |
| N | 1,865 | 1,865 | 1,865 | 1,865 | 1,865 | 1,865 |
| R2 | .02 | .12 | .01 | .10 | .14 | .17 |

Beta coefficients, standard errors in parentheses

* p<0.05, ** p<0.01, *** p<0.001

Source: Pairfam 11 and Corona survey

*Table S2*. Results from linear regression models for n=1,865 women and men predicting gender differences in levels of stress, lack of energy and loneliness before and during the Covid-19 pandemic by family type

|  | Stress | | Lack of Energy | | Loneliness | |
| --- | --- | --- | --- | --- | --- | --- |
| Variable | Before pandemic | Spring 2020 | Before pandemic | Spring 2020 | Before pandemic | Spring 2020 |
|  |  |  |  |  |  |  |
| Single parent | .32  (.20) | -.04  (.27) | .32  (.20) | -.46  (.27) | .97***  (.18) | .21 (.26) |
| Two parent family | .07  (.09) | .18*  (.08) | .04  (.09) | -.05  (.08) | .14  (.09) | -.08  (.08) |
| Single | -.07  (.11) | .04  (.10) | .14  (.11) | .06  (.10) | 1.12***  (.10) | .48***  (.10) |
| Childless couple (Reference) |  |  |  |  |  |  |
| Women (vs. men) | .06  (.09) | .14  (.09) | .17  (.09) | .14  (.09) | .24**  (.09) | .22**  (.08) |
| Woman*single parent | .18  (.22) | .33  (.29) | -.45*  (.23) | .77**  (.29) | -.21  (.21) | .02  (.28) |
| Woman*two parent family | -.02  (.11) | .08  (.11) | -.19  (.12) | .08  (.11) | -.12  (.11) | -.03  (.10) |
| Woman*single | .15  (.15) | .01  (.14) | -.27  (.15) | -.14  (.14) | -.38**  (.14) | -.11  (.14) |
| Woman*childless couple (Reference) |  |  |  |  |  |  |
| Pre-pandemic level of mental health |  | .29***  (.02) |  | .29***  (.02) |  | .29***  (.02) |
| University educated (vs. not) | .13**  (.05) | .01  (.04) | -.10*  (.05) | .01  (.04) | -.02  (.04) | -.07  (.04) |
| Birth cohort  1991-93 | .10  (.07) | -.13*  (.06) | -.02  (.07) | .05  (.06) | .07  (.06) | .08  (.06) |
| 1981-83 (Reference) |  |  |  |  |  |  |
| 1971-73 | -.13*  (.05) | -.17***  (.05) | -.14*  (.06) | -.14**  (.05) | -.04  (.05) | -.07  (.05) |
| N | 1,865 | 1,865 | 1,865 | 1,865 | 1,865 | 1,865 |
| R2 | .02 | .12 | .01 | .11 | .14 | .18 |

Beta coefficients, standard errors in parentheses

* p<0.05, ** p<0.01, *** p<0.001

Source: Pairfam 11 and Corona survey

### ***The emerging gender gap in mental health within two-parent families during the pandemic***

In this section, we report the supplementary analysis with the goal to grasp the origin of the emerging gender gap in mental health between women and men in two parent families during the pandemic. Increased childcare demands may have affected mothers and fathers differently due to gendered role practices. In our sample, 40% of mothers, compared to 14% fathers, in two-parent families reported that they assumed the entire responsibility for childcare during the spring lockdown. At the same time, almost twice as many men as women reported that they were sharing childcare duties with their partner to some extent. We thus suspected that unequal childcare demands could explain the mental health gap between mothers and fathers during the pandemic. We assessed the childcare burden by first taking into account the age of the youngest child, distinguishing between preschool age (i.e., under age six), primary school age (i.e., six to <10 years), and secondary school age (10 to 18 years)^[[1]](#footnote-1)^. We also sought to capture the childcare burden by the extent to which it conflicted with employment. For two-parent families, preschool childcare centers and schools were closed in March – May/June 2020, and access to emergency care was given only to families with at least one partner working in crucial infrastructure. In our sample, less than 10% of respondents reported that their children used such emergency care during the spring lockdown. The most prevalent work-related change in our sample was the proportion of respondents who switched from working at their workplace to working from home during the spring lockdown. This change was observed among almost half of our sample, and the gender differences were larger than in any other work-related change that the parents in our sample experienced (50% of fathers vs. 40% of mothers). We thus expected that work-family conflict levels might have been higher when performing paid work at home coincided with managing childcare and/or schooling at home. We also assumed that women might have been more affected than men might by these kinds of work-family conflicts, with potentially adverse mental health consequences. For mothers and fathers in two-parent families, we predicted each of the mental health outcomes by the set of predictors from the main models, and added a two-way interaction between the sex of the respondent and the age group of the youngest child. Figure S1 plots the results of this analysis (the results of the full regression models are presented in Table S3 below).

*Figure S1. Gender Differences in Mental Health among Mothers and Fathers in Two-parent-families by Age of Youngest Child before and during the Pandemic (n = 1,865)*

**

*Note: Models control for university degree (yes/no), birth cohort. Models on 2020 data additional control for pre-pandemic level of mental health in corresponding dimension.*

*Source: Pairfam Wave 11 (2018/19) and Corona Survey*

For the pre-pandemic sample, taking into account the age of the youngest child does not explain the gender mental health gap in loneliness (which was the only gender difference we found). During the pandemic, for stress and loneliness, but not for a lack of energy, we found a clear gender gap in mental health to the disadvantage of mothers when the youngest child was preschool age, while the differences among mothers and fathers of school-aged children were smaller (preschool) or non-existent (secondary). However, as the main gender effect remained statistically significant, we cannot entirely explain the gender gap in the parents’ mental health. In a second set of models we added a three-way interaction between sex, age of youngest child and working from home. Figure S2 plots the results of this analysis (the results of the full regression models are presented in Table S4 below).

*Figure S2. Gender Differences in Mental Health among Mothers and Fathers in Two-parent-families by Age of Youngest Child and Working from Home (yes/no) during the Pandemic (n=847)*

*Note: Models control for university degree (yes/no), birth cohort and pre-pandemic level of mental health in corresponding dimension.*

*Source: Pairfam Wave 11 (2018/19) and Corona Survey*

For stress, but not energy and loneliness, we find clear evidence that the gender stress gap in two parent families is explained by mothers of pre-school aged children who work from home reporting significantly higher stress levels than their female counterparts who do not work from home (*Figure S2*). The stress level of mothers with a pre-school age child who did not work from home in spring 2020 is equivalent to the predicted stress level of a father who did work from home in the same period. These findings suggest that mothers’ mental health is more negatively affected by work-family-conflict during the spring lockdown in Germany.

*Table S3*. Results from linear regression models for n=847 mothers and fathers in two-parent families predicting gender differences in levels of stress, lack of energy, and loneliness before and during the COVID-19 pandemic by age group of the youngest child

|  | Stress | | Lack of energy | | Loneliness | |
| --- | --- | --- | --- | --- | --- | --- |
|  | Before pandemic | Spring 2020 | Before pandemic | Spring 2020 | Before pandemic | Spring 2020 |
| Women (vs. men) | -.02  (.09) | .26**  (.08) | -.04  (.09) | .13  (.09) | .21**  (.07) | .22**  (.08) |
| Preschool (Reference) |  |  |  |  |  |  |
| Primary school | -.27  (.15) | -.06  (.15) | -.20  (.16) | .04  (.15) | .20  (.13) | -.01  (.13) |
| Secondary school | -.21  (.14) | -.20  (.14) | -.17  (.15) | .04  (.14) | -.02  (.12) | -.15  (.13) |
| Woman*preschool (Reference) |  |  |  |  |  |  |
| Woman*primary school | .25  (.18) | .07  (.18) | .09  (.19) | .00  (.18) | -.21  (.16) | -.14  (.16) |
| Woman*secondary school | -.04  (.16) | -.19  (.16) | .07  (.17) | .09  (.16) | -.11  (.14) | -.15  (.15) |
| Pre-pandemic level of mental health |  | .29***  (.03) |  | .25***  (.03) |  | .25***  (.03) |
| University-educated (vs. not) | .11  (.07) | .03  (.07) | -.14*  (.07) | -.02  (.07) | -.04  (.06) | -.09  (.06) |
| Birth cohort  1991-93 | .04  (.17) | -.26  (.15) | .01  (.18) | .15  (.16) | .01  (.15) | .34*  (.14) |
| 1981-83 (Reference) |  |  |  |  |  |  |
| 1971-73 | -.00  (.09) | -.10  (.09) | -.12  (.09) | -.25**  (.09) | .05  (.08) | .01  (.08) |
| N | 847 | 847 | 874 | 847 | 847 | 847 |
| R2 | .06 | .13 | .09 | .08 | .01 | .10 |

Beta coefficients, standard errors in parentheses

* p<0.05, ** p<0.01, *** p<0.001

Source: Pairfam Corona survey

*Table S4.* Results from linear regression models for n=847 mothers and fathers in two-parent families predicting gender differences in levels of stress, lack of energy, and loneliness during the COVID-19 pandemic by age group of the youngest child and working from home

|  | Stress | Lack of energy | Loneliness |
| --- | --- | --- | --- |
| Women (vs. men) | .18  (.12) | .18  (.12) | .29**  (.11) |
| Worked from home (vs. not) | .16  (.13) | .22  (.13) | .00  (.11) |
| Preschool (Reference) |  |  |  |
| Primary school | -.12  (.22) | -.07  (.22) | -.04  (.20) |
| Secondary school | -.16  (.19) | .10  (.20) | -.24  (.17) |
| Women*worked from home (vs. not) | .19  (.17) | -.08  (.17) | -.18  (.15) |
| Worked from home (vs. not)*preschool (Reference) |  |  |  |
| Worked from home (vs. not)* primary school | .06  (.29) | .16  (.29) | .06  (.26) |
| Worked from home (vs. not)*secondary school | -.11  (.25) | -.15  (.26) | .19  (.23) |
| Women *preschool (Reference) |  |  |  |
| Women*primary school | .28  (.26) | .08  (.26) | .06  (.26) |
| Women*secondary school | -.10  (.22) | .06  (.22) | .19  (.23) |
| Women*worked from home*preschool (Reference) |  |  |  |
| Women*worked from home*primary school | -.40  (.36) | -.11  (.37) | .16  (.33) |
| Women*worked from home *secondary school | -.21  (.33) | .03  (.33) | .22  (.30) |
| Pre-pandemic level of mental health indicator | .28***  (.03) | .24***  (.03) | .25***  (.03) |
| University-educated (vs. not) | -.04  (.07) | -.07  (.07) | -.09  (.06) |
| Birth cohort  1991-93 | -.23  (.17) | .19  (.17) | .35*  (.15) |
| 1981-83 (Reference) |  |  |  |
| 1971-73 | -.08  (.09) | -.24**  (.09) | -.01  (.08) |
| N | 847 | 847 | 847 |
| R2 | .14 | .09 | .11 |

***Robustness analysis***

In this section we address potential bias of the results presented here arising from non-participation in the optional, so-called “Covid-19-survey” that was fielded during the pandemic in spring 2020. From 9,640 eligible Pairfam panelists (i.e., the gross sample of the regular panel wave 12 whose fieldwork was interrupted by the pandemic), 33 percent particpated in the optional web survey. Given the scope of the present study, we put additional selection criteria to obtain our analytical sample of n=1,865 women and men (reported in the main paper).

As a robustness analyses, we applied inverse probability weights to our regression models. In order to obtain these weights, we predicted participation in the optional websurvey in 2020 using logit regression models including a set of characteristics known to predict panel attrition. These predictores were taken from the pre-pandemic wave of data collection (2018/19) and covered (a) sociodemographic characterstics (i.e., gender, age, education, partnership status, migration status, household size; (b) geographic context (i.e., urban vs. rural, East Germany vs. West Germany); (c) economic deprivation; (d) personality (i.e., self esteem, trust; (e) physical health and (f) interview(er) characteristics (i.e., number of contacts, gender, age and education of interviewer, duration of interview.

Table S5 shows the results of this analysis. Women, younger cohort members, highly educated and non-migrants where more likely to participate while household size did not predict participation in the Corona survey. Residents of urban areas and West Germans were more likely to participate while higher economic deprivation reduced the probability to participate. Respondents with lower self-esteem and higher trust as well as physically healthier respondents were more likely to participate. Among different kinds of characteristics of the interview and the interviewer in 2018/2019, only a higher number of contacts was associated with a lower probability of participating in the Corona survey.

*Table S5. Results from logistic regression models for n=9,251 women and men predicting participation in the Corona survey in spring 2020*

|  | β | SE |
| --- | --- | --- |
| ***Socio-demographic characteristics*** |  |  |
| Women (vs. men) | .315*** | .048 |
| Birth cohort  1991-93 (Reference) |  |  |
| 1981-83 | .422*** | .066 |
| 1971-73 | .575*** | .075 |
| 2001-03 | 1.084*** | .120 |
| Single (vs. not) | .091 | .061 |
| Education (in years) | .051*** | .008 |
| Migrant (vs. not) | -.507*** | .061 |
| Household size | .038 | .065 |
| ***Geographic context*** |  |  |
| Urban (vs. rural) | .127* | .050 |
| East (vs. West) Germany | -.213*** | .057 |
| ***Economic deprivation*** | -.147 | .023 |
| ***Personality*** |  |  |
| Self esteem | -.073** | .028 |
| Trust | .222*** | .032 |
| ***Physical health*** | .012*** | .003 |
| ***Interview(er) characteristics*** |  |  |
| Number of contacts | -.031*** | .009 |
| Interviewer female (vs. male) | .056 | .047 |
| Interviewer’s age | -.003 | .016 |
| Interviewer’s low education (vs. not) | .019 | .049 |
| Interview duration | .032 | .041 |
| Interview duration squared | -0.00 | .002 |
| N | 9,251 |  |
| R2 | 0.043 |  |

Based on this model, we estimated inverse probability weights for each responsent and applied these to the models presented in the main paper. The results are presented below in Tables S6 and S7. The conclusions drawn from these weighted robustness analyses are virtually identicial to the results based on unweighted models presented in the main paper.

*Table S6*. Results from linear regression models applying inverse probability weights for n=1,865 women and men predicting gender differences in levels of stress, lack of energy and loneliness before and during the Covid-19 pandemic by family type

|  | Stress | | Lack of Energy | | Loneliness | |
| --- | --- | --- | --- | --- | --- | --- |
| Variable | Before pandemic | Spring 2020 | Before pandemic | Spring 2020 | Before pandemic | Spring 2020 |
| Single parent | .40***  (.10) | .23*  (.10) | .02  (.10) | .16  (.10) | .76***  (.09) | .26*  (.10) |
| Two parent family | .01  (.06) | .23***  (.06) | -.10  (.07) | .02  (.06) | .01  (.06) | -.04  (.06) |
| Single | -.01  (.07) | .05  (.07) | .02  (.07) | .01  (.07) | .88***  (.07) | .46***  (.07) |
| Childless couple (Reference) |  |  |  |  |  |  |
| Women (vs. men) | .13**  (.05) | .18***  (.04) | .08  (.05) | .20***  (.05) | .15***  (.04) | .18***  (.04) |
| Pre-pandemic level of mental health |  | .29***  (.02) |  | .29*** (.02) |  | .29***  (.02) |
| University educated (vs. not) | .18*** (.05) | -.01  (.04) | -.06  (.05) | -.01  (.04) | .00  (.04) | -.11*  (.04) |
| Birth cohort  1991-93 | .01  (.06) | -.11  (.06) | -.07  (.06) | .10  (.06) | .02  (.06) | .08  (.06) |
| 1981-83 (Reference) |  |  |  |  |  |  |
| 1971-73 | -.09  (.06) | -.17***  (.05) | -.13*  (.06) | -.12*  (.05) | -.01  (.05) | -.07  (.05) |
| N | 1,865 | 1,865 | 1,865 | 1,865 | 1,865 | 1,865 |
| R2 | .02 | .12 | .01 | .10 | .14 | .16 |

Beta coefficients, standard errors in parentheses

* p<0.05, ** p<0.01, *** p<0.001

Source: Pairfam 11 and Corona survey

*Table S7*. Results from linear regression models applying inverse probability weights for n=1,865 women and men predicting gender differences in levels of stress, lack of energy and loneliness before and during the Covid-19 pandemic by family type

|  | Stress | | Lack of Energy | | Loneliness | |
| --- | --- | --- | --- | --- | --- | --- |
| Variable | Before pandemic | Spring 2020 | Before pandemic | Spring 2020 | Before pandemic | Spring 2020 |
|  |  |  |  |  |  |  |
| Single parent | .18  (.17) | .02  (.23) | .19  (.18) | -.44  (.23) | .99***  (.16) | .17  (.23) |
| Two parent family | -.03  (.09) | .19*  (.08) | -.03  (.09) | -.03  (.08) | .06  (.08) | .00  (.08) |
| Single | -.12  (.10) | .03  (.09) | .13  (.10) | .03  (.10) | 1.06***  (.10) | .51***  (.10) |
| Childless couple (Reference) |  |  |  |  |  |  |
| Women (vs. men) | .04  (.09) | .12  (.08) | .20*  (.09) | .14  (.08) | .28**  (.09) | .23**  (.08) |
| Woman*single parent | .32  (.21) | .26  (.25) | -.27  (.21) | .75**  (.26) | -.35  (.19) | .08  (.25) |
| Woman*two parent family | .08  (.11) | .08  (.10) | -.13  (.12) | .09  (.10) | -.10  (.11) | -.08  (.10) |
| Woman*single | .22  (.15) | .03  (.14) | -.22  (.15) | -.06  (.14) | -.35*  (.14) | -.09 (.14) |
| Woman*childless couple (Reference) |  |  |  |  |  |  |
| Pre-pandemic level of mental health |  | .29***  (.02) |  | .28***  (.02) |  | .27***  (.02) |
| University educated (vs. not) | .17***  (.05) | -.01  (.04) | -.05  (.05) | -.00  (.04) | .01  (.04) | -.11*  (.04) |
| Birth cohort  1991-93 | .01  (.06) | -.10  (.06) | -.07  (.06) | .11  (.06) | .02  (.06) | .08  (.06) |
| 1981-83 (Reference) |  |  |  |  |  |  |
| 1971-73 | -.10  (.06) | -.16**  (.05) | -.13*  (.06) | -.12*  (.05) | -.00  (.05) | -.07  (.05) |
| N | 1,865 | 1,865 | 1,865 | 1,865 | 1,865 | 1,865 |
| R2 | .03 | .12 | .01 | .11 | .14 | .16 |

Beta coefficients, standard errors in parentheses

* p<0.05, ** p<0.01, *** p<0.001

Source: Pairfam 11 and Corona survey

1. In the coronavirus questionnaire, the age of child was not measured, and by deriving it from the age of the youngest child at wave 11, we would miss children born between 2018/19 and 2020. Thus, we derived the age group of the youngest child in three categories based on the respondents’ answers to questions about the availability of preschool child care during the pandemic (respondents were invited to answer only if they had children in that age group), and their valid responses to a question on the grade level of their youngest child enrolled in school. We thus distinguished three age groups of a respondent’s youngest child: preschool (i.e., below age six), primary school (i.e., age six to 10) and secondary school (i.e., age 11 – 18). For the sake of the comparability of our results during the pandemic with the measures one year prior to the pandemic, we applied the same classification scheme for the youngest child in the pre-pandemic wave, although we had the exact age available (but not the grade level). Consequently, we might have misclassified some children in the pre-pandemic wave as being at a higher-grade level than they were in reality. This is because in Germany, primary school entry takes place in August, and children who are born in September – December enter primary school at age seven (i.e., in the following year). [↑](#footnote-ref-1)
